# Idiosyncratic Drug-Induced Liver Injury and Amoxicillin–Clavulanate: Spotlight on Gut Microbiota, Fecal Metabolome and Bile Acid Profile in Patients

**DOI:** 10.3390/ijms25136863

**Published:** 2024-06-22

**Authors:** Sara Román-Sagüillo, Raisa Quiñones Castro, María Juárez-Fernández, Polina Soluyanova, Camilla Stephens, Mercedes Robles-Díaz, Francisco Jorquera Plaza, Javier González-Gallego, Susana Martínez-Flórez, María Victoria García-Mediavilla, Esther Nistal, Ramiro Jover, Sonia Sánchez-Campos

**Affiliations:** 1 Instituto Universitario de Biomedicina (IBIOMED), Universidad de León, 24071 León, Spain; sroms@unileon.es (S.R.-S.); mjuarf@unileon.es (M.J.-F.); fjorqueraplaza@gmail.com (F.J.P.); jgonga@unileon.es (J.G.-G.); smarf@unileon.es (S.M.-F.); mvgarm@unileon.es (M.V.G.-M.); menisg@unileon.es (E.N.); 2 Servicio de Aparato Digestivo, Complejo Asistencial Universitario de León, 24008 León, Spain; rquinones@saludcastillayleon.es; 3 Centro de Investigación Biomédica en Red de Enfermedades Hepáticas y Digestivas (CIBERehd), Instituto de Salud Carlos III, 28029 Madrid, Spain; cstephens@uma.es (C.S.); mercedesroblesdiaz@hotmail.com (M.R.-D.); ramiro.jover@uv.es (R.J.); 4 Unidad Mixta de Investigación en Hepatología Experimental, IIS Hospital La Fe, 46026 Valencia, Spain; polina.soluyanova@uv.es; 5 Departamento de Bioquímica y Biología Molecular, Universidad de Valencia, 46010 Valencia, Spain; 6 Unidad de Gestión Clínica de Aparato Digestivo y Servicio de Farmacología Clínica, Instituto de Investigación Biomédica de Málaga-IBIMA Plataforma BIONAND, Hospital Universitario Virgen de la Victoria, Facultad de Medicina, Universidad de Málaga, 29010 Málaga, Spain

**Keywords:** drug-induced liver disease, amoxicillin–clavulanate, gut microbiota, bile acids, fecal metabolome

## Abstract

Several hepatic disorders are influenced by gut microbiota, but its role in idiosyncratic drug-induced liver injury (iDILI), whose main causative agent is amoxicillin–clavulanate, remains unknown. This pioneering study aims to unravel particular patterns of gut microbiota composition and associated metabolites in iDILI and iDILI patients by amoxicillin–clavulanate (iDILI-AC). Thus, serum and fecal samples from 46 patients were divided into three study groups: healthy controls (n = 10), non-iDILI acute hepatitis (n = 12) and iDILI patients (n = 24). To evaluate the amoxicillin–clavulanate effect, iDILI patients were separated into two subgroups: iDILI non-caused by amoxicillin–clavulanate (iDILI-nonAC) (n = 18) and iDILI-AC patients (n = 6). Gut microbiota composition and fecal metabolome plus serum and fecal bile acid (BA) analyses were performed, along with correlation analyses. iDILI patients presented a particular microbiome profile associated with reduced fecal secondary BAs and fecal metabolites linked to lower inflammation, such as dodecanedioic acid and pyridoxamine. Moreover, certain taxa like *Barnesiella*, *Clostridia UCG-014* and *Eubacterium* spp. correlated with significant metabolites and BAs. Additionally, comparisons between iDILI-nonAC and iDILI-AC groups unraveled unique features associated with iDILI when caused by amoxicillin–clavulanate. In conclusion, specific gut microbiota profiles in iDILI and iDILI-AC patients were associated with particular metabolic and BA status, which could affect disease onset and progression.

## 1. Introduction

Drug-induced liver injury (DILI), defined as liver damage caused by complex adverse reactions to medication [1], is considered the fifth cause of liver-related death worldwide [2] and one of the main causes of acute liver failure (ALF) in the Western world [3]. However, this disease probably has a higher impact on human health, as its actual worldwide prevalence is likely underestimated due to difficulties associated with the diagnosis [4].

Non-predictable DILI based on drug dose and pharmacological action is defined as idiosyncratic DILI (iDILI), which has become the most challenging form of this disease [1]. iDILI is one of the most common reasons for drug development attrition, pharmaceutical regulatory decisions and post-marketing global drug withdrawal from the market, resulting not only in a high economic expenditure but also in a large impact on healthcare systems [4]. Amoxicillin–clavulanate (AC), one of the most prescribed antibiotics in Europe and the United States [5,6], has been considered a leading cause of iDILI [3,4,7] when it is orally administered. Although the incidence of iDILI caused by AC (DILI-AC) is known to be higher than iDILI caused by amoxicillin alone [6], the worldwide incidence of this specific condition is still undetermined.

Regarding the therapeutic management of iDILI, there is no specific pharmacological therapy to overcome this condition [8]. The absence of a standardized treatment protocol, interindividual variations in its development and lack of knowledge about the specific pathological mechanisms of iDILI make this condition difficult to manage. Currently, withdrawal of the causative drug is the main procedure, often leading to a spontaneous resolution [4]. However, severe iDILI cases can progress to ALF, with liver transplantation and palliative care being the main therapeutic strategies [8].

Over the last few years, the study of the gut microbiota has provided useful information about the specific mechanisms involved in the onset and development of many pathologies, including liver-related diseases such as non-alcoholic fatty liver disease and steatohepatitis [9,10,11,12,13,14]. In fact, it is widely recognized that the gut microbiota plays a key role in the development of many hepatic diseases, mainly through the anatomical connection between the gut and the liver, the gut–liver axis [15]. Moreover, the specific differences in gut microbiota composition could influence the metabolization of oral drugs and the consequent interindividual differences in the susceptibility to drug hepatotoxicity [8]. Herein, considering that the pathophysiological process of iDILI is influenced by a complex combination of intrinsic and extrinsic factors, the gut microbiota and its regulatory role in host metabolism have been pointed out as a key element involved in the disease [16,17]. In relation to this, Chu et al. (2023) suggested a possible mechanism of gut microbiota affecting DILI based on an altered permeability of the intestinal barrier caused by gut dysbiosis, promoting the translocation of certain microorganisms and resulting in higher levels of inflammatory cytokines as well as increased oxidative stress through the production of reactive oxygen species [16].

Bile acids (BAs) are one of the most relevant metabolites related not only to the gut microbiota but also to the development and progression of different forms of DILI. The deconjugation of primary BAs and the consequent production of secondary BAs are processes performed by the gut microbiota [18], and high concentrations of total BAs have been considered a marker of liver injury [19]. Additionally, the emulsifying detergent properties of these molecules could compromise the cell membrane integrity, causing cytotoxic effects and promoting DILI development [19]. Other metabolites, such as short-chain fatty acids (SCFAs), are closely related to the modulation of the inflammatory and immune status, and altered gut microbiota in iDILI patients may change the content of these metabolites, participating in the pathogenesis of DILI [16]. For all of that, a better understanding of the role of gut microbiota in the onset and progression of DILI could provide new strategies to manage or even prevent this disease.

Currently, there is no general preclinical model of iDILI that enables the study of this condition [20]. Furthermore, due to the low incidence of patients with iDILI, specific clinical trials are also not a feasible resource to investigate this disease. Moreover, the link between the gut microbiota and iDILI has been poorly investigated in patients. In the present study, we aimed to elucidate the existence of particular patterns of gut microbiota composition in patients with iDILI and iDILI-AC. Thus, a specific compositional and functional gut microbiota profile linked to patients with iDILI and iDILI-AC was identified. These findings point out an association between DILI and gut microbiota metabolism through changes in fecal metabolome and BA profile, which may contribute to the identification of possible disease hallmarks as feasible biomarkers for early detection and diagnosis.

## 2. Results

**Figure 1:**
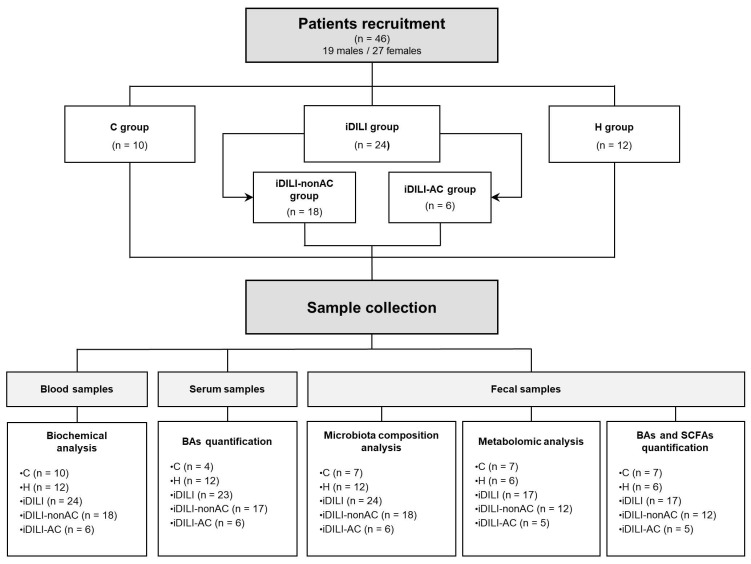
Experimental design. C, healthy control; H, non-iDILI acute hepatitis; BAs, bile acids; iDILI, idiosyncratic drug-induced liver injury; iDILI-AC, idiosyncratic drug-induced liver injury caused by amoxicillin–clavulanate; iDILI-nonAC, idiosyncratic drug-induced liver injury caused by compounds other than amoxicillin–clavulanate; SCFAs, short-chain fatty acids.

For a better understanding of the results, the distribution of the study groups is described in detail in Figure 1.

### 2.1. Hematological and Biochemical Alterations Observed in Patients with Idiosyncratic Drug-Induced Liver Injury

Hematological and serum biochemical analyses were performed to determine differences among the healthy control (C), iDILI and non-iDILI acute hepatitis (H) groups, especially those associated with the iDILI group (Table 1). 

Parameters related to liver damage and impaired hepatic function, such as alanine aminotransferase (ALT), aspartate aminotransferase (AST), alkaline phosphatase (ALP), gamma-glutamyl transferase (GGT) and total bilirubin (TBL) were significantly incremented, while the albumin concentration was significantly reduced in the iDILI and H groups compared to the C group, confirming the presence of hepatic damage and liver dysfunction (Table 1). In this sense, ALT and AST activities were significantly higher in H patients compared to iDILI patients, and a similar non-significant trend was observed in GGT activity. Moreover, neutrophil levels were significantly higher in the H group with regard to the other two groups as well as TBL and the International Normalized Ratio (INR), which were increased in the H group compared to the C group, suggesting a worsened hepatocellular injury in this group (Table 1).

### 2.2. Analysis of Fecal Gut Microbiota Composition Shows a Specific Profile Associated with Idiosyncratic Drug-Induced Liver Injury

**Figure 2:**
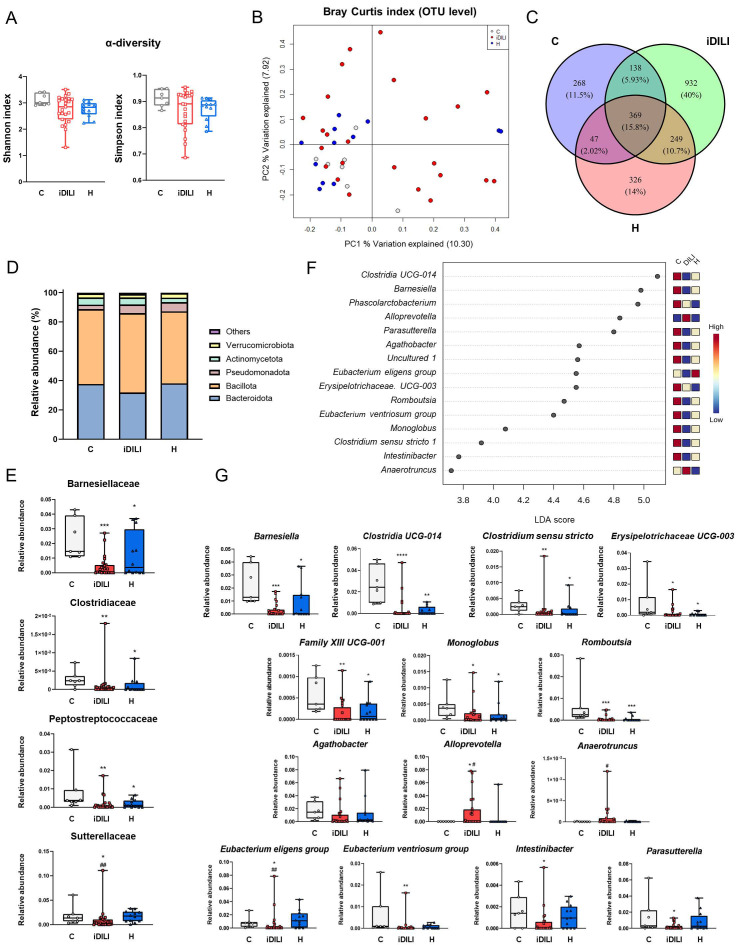
Differences in gut microbiota composition associated with idiosyncratic drug-induced liver injury. (**A**) α-diversity measured by the Shannon and Simpson index. (**B**) Principal coordinates analysis (PCoA) plot representing β-diversity based on the Bray–Curtis similarity index at the operational taxonomic unit (OTU) level. The percentage of the total variance explained is indicated in parentheses on each axis. (**C**) Venn diagram at the OTU level. (**D**) Differences in the relative abundance at the phylum level. (**E**) Boxplots representing differences in the relative abundance at the family level. (**F**) Linear discriminant analysis (LDA) at the genus level. *p*-value cutoff was settled on 0.1. Log LDA score was settled on 2.0. (**G**) Boxplots representing differences in the relative abundance at the genus level. * *p* < 0.05; ** *p* < 0.01; *** *p* < 0.001; **** *p* < 0.0001 vs. C; ^#^ *p* < 0.05; ^##^ *p* < 0.01 vs. H by a non-parametric Kruskal–Wallis test followed by the Mann–Whitney U-Test. C, healthy control group (n = 7); iDILI, idiosyncratic drug-induced liver disease group (n = 24); H, non-iDILI acute hepatitis group (n = 12); LDA, linear discriminant analysis; OUT, operational taxonomic unit.

Fecal samples were analyzed in order to determine the gut microbial profile associated with iDILI. Gut microbiota sequencing confirmed the presence of gut dysbiosis in the H and iDILI groups compared to the C group. iDILI and H groups presented slightly reduced Shannon and Simpson α-diversity indexes compared to the C group (Figure 2A).

β-diversity measurements were performed to assess differences in global bacterial composition among the three groups using principal coordinates analysis (PCoA) based on the Bray–Curtis index at the operational taxonomic unit (OTU) level. This analysis revealed that bacterial communities of the healthy controls and the H group tended to cluster, whereas iDILI patients were spread along the PC1 (10.3%) and PC2 (7.92%) (PERMANOVA *p* = 0.2105) (Figure 2B). To identify possible characteristic profiles associated with each group, a Venn diagram was performed at the OTU level. Thus, 369 out of 2329 OTUs (15.8%) were shared among all samples, while 10.7% were shared between the iDILI and H groups. Additionally, 40% of all the identified OTUs were found exclusively in the iDILI group (Figure 2C). 

The analysis of the bacterial taxa showed significant differences among groups. At the phylum level, the iDILI group was characterized by an increase in the relative abundance of Bacillota and by a reduction in the relative abundance of Bacteroidota and Verrucomicrobiota compared to the other groups, although these differences did not reach statistical significance (Figure 2D). At the family level, a significantly lower relative abundance of Barnesiellaceae, Clostridiaceae and Peptostreptococcaceae was observed in the iDILI and H groups compared to the C group (Figure 2E). In addition, the H group showed a reduced relative abundance of Acidaminococcaceae compared to the C group (Supplementary Material Figure S1A), whereas iDILI patients presented a lower relative abundance in Sutterellaceae with respect to the C and H groups (Figure 2E). 

Moreover, a linear discriminant analysis (LDA) of the 15 most representative genera was performed to identify possible profiles related to each group (Figure 2F). Both the H and iDILI groups shared some taxa, although an individual characteristic profile in each group was also observed at the genus level. Herein, a significant reduction in the relative abundance of *Barnesiella*, *Clostridia UCG-014*, *Clostridium sensu stricto 1*, *Erysipelotrichaceae UCG-003*, *Family XIII UCG-001*, *Monoglobus* and *Romboutsia* was observed in the iDILI and H groups compared to healthy patients (Figure 2G). Regarding the specific profile observed in the iDILI group at the genus level, a higher relative abundance of *Alloprevotella* and a lower relative abundance of *Eubacterium eligens group* were identified in comparison with both other two groups. Moreover, in terms of the differences with the C group, *Anaerotruncus* was increased while *Agathobacter*, *Eubacterium ventriosum group*, *Intestinibacter* and *Parasutterella* genera presented an opposite pattern in the iDILI group (Figure 2G). Contrarily, the H profile observed at the genus level was characterized by a significantly lower relative abundance of *Phascolarctobacterium* and *Eubacterium coprostanoligenes group* compared to the C group and a noticeably higher relative abundance of *Lachnospiraceae UCG-004* compared to both the C and iDILI groups (Supplementary Figure S1A). 

### 2.3. Idiosyncratic Drug-Induced Liver Injury Is Characterized by a Specific Fecal Metabolome and Bile Acid Profile

**Figure 3:**
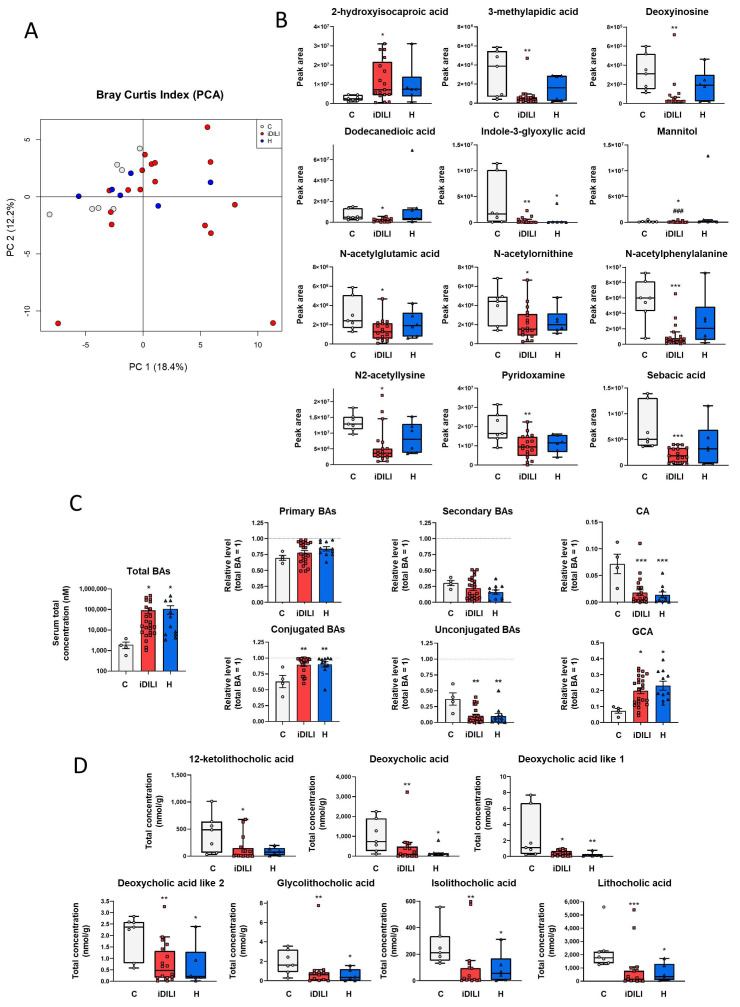
Differences in fecal gut metabolome related to idiosyncratic drug-induced liver injury. (**A**) Principal component analysis (PCA) based on the Bray–Curtis index showing the differences in the fecal metabolomic profile. The percentage of the total variance explained is indicated in parenthesis in XY axes. (**B**) Boxplots representing non-targeted metabolites expressed as the peak area of detection. * *p* < 0.05; ** *p* < 0.01; *** *p* < 0.001 vs. C; ^###^ *p* < 0.001 vs. H by a non-parametric Kruskal–Wallis test followed by the Mann–Whitney U-Test. (**C**) Total bile acid concentration and relative levels of primary, secondary, conjugated and unconjugated bile acids in serum. Relative levels of glycocholic and cholic acid. The dashed line represents total bile acid levels relativized to one. * *p* < 0.05; ** *p* < 0.01; *** *p* < 0.001 vs. C by one-way ANOVA and data are presented as mean ± SEM. (**D**) Boxplots showing significant fecal bile acids. * *p* < 0.05; ** *p* < 0.01; *** *p* < 0.001 vs. C by a non-parametric Kruskal–Wallis test followed by the Mann–Whitney U-Test. BAs, bile acids; C, healthy control group (Figure 2A,B,D: n = 7; Figure 2C: n = 4); CA, cholic acid; GCA, glycocholic acid; iDILI, idiosyncratic drug-induced liver disease group (Figure 2A,B,D: n = 17; Figure 2C: n = 23); H, non-iDILI acute hepatitis group (Figure 2A,B,D: n = 6; Figure 2C: n = 12).

Non-targeted fecal metabolomic analysis of semi-polar compounds showed a characteristic profile related to iDILI. First, a principal component analysis (PCA) was performed to study the effect associated with iDILI on the metabolome composition of those patients, highlighting a significantly higher dispersion of iDILI samples in comparison with the C and H groups along principal component 1 (PC1) (18.4%) (PERMANOVA *p* = 0.0332) (Figure 3A). 

Moreover, as shown in Figure 3B, the abundance of the metabolites 3-methylapidic acid, deoxyinosine, dodecanedioic acid, mannitol, N-acetylglutamic acid, N-acetylornithine, N-acetylphelylalanine, N2-acetyllysine, pyridoxamine and sebacic acid was significantly reduced in the iDILI group, whereas 2-hydroxyisocaproic acid showed the opposite pattern compared to both the H and C groups. Additionally, H and iDILI patients shared a significantly lower detection of indole-3-glyoxylic acid compared to the C group (Figure 3B). In relation to lipid and energetic metabolism, fecal quantification of the main SCFAs, acetic, butyric and propionic acids, was performed, although no significant differences among the three groups were observed (Supplementary Figure S1B).

Furthermore, considering the relationship between iDILI development and BA metabolism, the quantification of these compounds was performed in both serum and fecal samples. First, serum BA quantification showed a higher concentration of total BAs in the iDILI and H groups compared to the C group (Figure 3C). In addition, conjugated BAs such as glycocholic acid presented a significantly higher concentration in these groups, whereas a significant reduction was identified in the unconjugated BAs such as cholic acid (Figure 3C). 

In this sense, iDILI and H patients also showed a particular BA composition pattern in feces, characterized by a reduction in the concentration of secondary BA-derived metabolites, such as deoxycholic acid, deoxycholic acid like 1 and 2, glycolithocholic, isolithocholic and lithocholic acids compared to healthy controls (Figure 3D). Moreover, a significantly lower fecal concentration of 12-ketolithocholic acid was identified only in the iDILI group (Figure 3D).

### 2.4. Correlations among Biochemical Parameters, Gut Microbiota Composition, Fecal Metabolome and Fecal and Serum Bile Acid Profiles in Patients with Idiosyncratic Drug-Induced Liver Injury

A correlation analysis was performed to identify possible links between iDILI development and gut microbiota composition and functionality. Therefore, correlation analyses among biochemical parameters, gut microbiota composition, fecal metabolome and fecal and serum BA profiles were performed.

**Figure 4:**
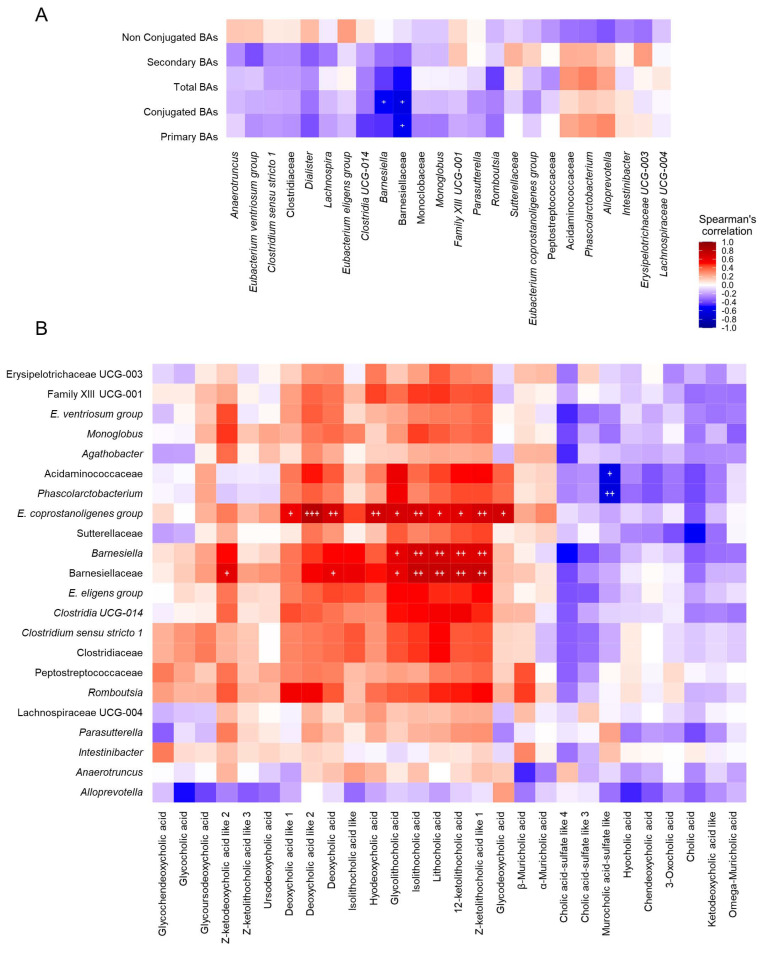
Correlation analysis among gut microbiota composition, fecal metabolome and fecal and serum bile acid profiles. (**A**) Heatmap of correlations between serum bile acids and gut microbiota composition. (**B**) Heatmap of correlations between gut microbiota composition and fecal bile acids. Each square represents the Spearman’s correlation coefficient (*p* < 0.05). Red and blue cells represent positive and negative correlations, respectively. White crosses designate the level of significance. ^+^ *p* < 0.05; ^++^ *p* < 0.01; ^+++^ *p* < 0.001. C, healthy control group (Figure 3A: n = 4; Figure 3B: n = 7); iDILI, idiosyncratic drug-induced liver disease group (Figure 3A: n = 23; Figure 3B: n = 17); H, non-iDILI acute hepatitis group (Figure 3A: n = 12; Figure 3B: n = 6).

First, serum and fecal BAs showed significant correlations with the identified gut microbiota profile. Herein, the Barnesiellaceae family was negatively correlated with serum primary and conjugated BAs, while the *Barnesiella* genus was negatively correlated only with serum conjugated BAs (Figure 4A). 

**Figure 5:**
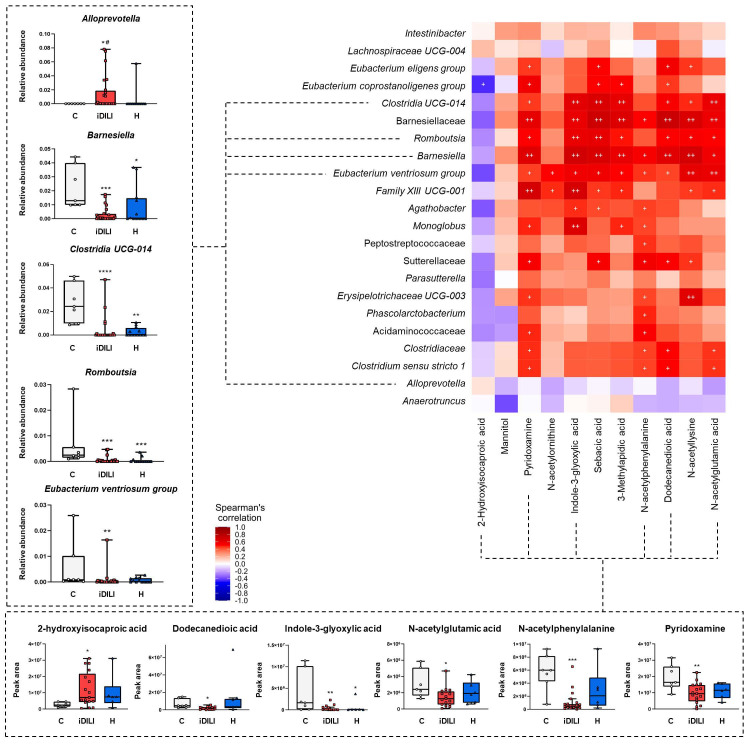
Correlation analysis of gut microbiota composition and metabolomic profile. Each square represents the Spearman’s correlation coefficient (*p* < 0.05). Red and blue cells represent positive and negative correlations, respectively. White crosses designate the level of significance. ^+^ *p* < 0.05; ^++^ *p* < 0.01. Remarkable bacterial taxa and fecal metabolites are represented in boxplots. * *p* < 0.05; ** *p* < 0.01; *** *p* < 0.001; **** *p* < 0.0001 vs. C; ^#^ *p* < 0.05 vs. H by a non-parametric Kruskal–Wallis test followed by the Mann–Whitney U-Test. C, healthy control group (n = 7); iDILI, idiosyncratic drug-induced liver disease group (n = 17); H, non-iDILI acute hepatitis group (n = 6).

With regard to gut microbiota composition and fecal metabolomic profile, a specific pattern of significant correlations was observed. In this sense, most of the correlations identified between fecal metabolites and different bacterial taxa were positive, highlighting those established between dodecanedioic acid, indole-3-glyoxylic acid, N-acetylglutamic acid, N-acetylphenylalanine, pyridoxamine, sebacic acid and 3-methyladipic acid and the genera *Barnesiella, Clostridia UCG014* and *Romboutsia*. Moreover, 2-hydroxyisocaproic acid was the only fecal metabolite that presented a slight negative correlation with the gut microbiota profile, which was significant for the *Eubacterium coprostanoligenes group* genus. Additionally, although no statistical significance was noticed, the *Alloprevotella* and *Anaerotruncus* genera showed a completely different correlation profile with the fecal metabolites that could be linked to iDILI development (Figure 5). 

### 2.5. Idiosyncratic Drug-Induced Liver Injury Caused by Amoxicillin–Clavulanate Administration Is Characterized by a Specific Pattern of Gut Microbiota Composition Linked to a Particular Metabolome and Bile Acid Profile

Considering that amoxicillin–clavulanate is one of the most frequent drugs causing iDILI and to find a possible specific profile linked to iDILI-AC, a comparison between iDILI-nonAC and iDILI-AC patients, following the same analysis performed in the study of the C, iDILI and H groups, was carried out. First, as mentioned above, no significant differences in hematological and biochemical parameters between iDILI-nonAC and iDILI-AC were observed (Supplementary Table S1).

**Figure 6:**
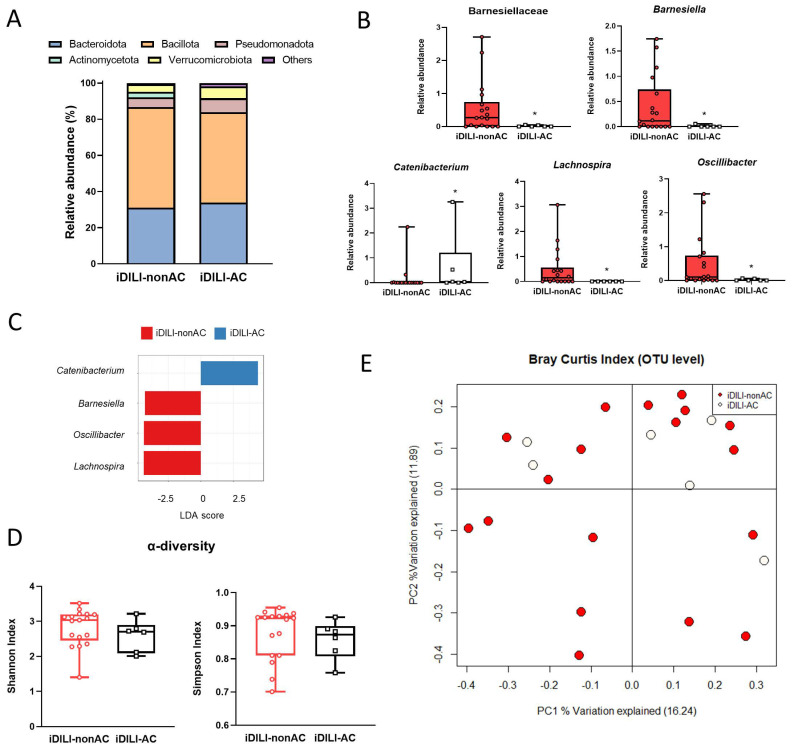
Differences in patients with idiosyncratic drug-induced liver injury caused by amoxicillin–clavulanate in gut microbiota composition, compared with iDILI-nonAC. (**A**) Differences in relative abundance at the phylum level. (**B**) Differences in relative abundance at the family and genus levels. (**C**) Linear discriminant analysis (LDA) at the genus level. *p*-value cutoff was settled on 0.1. Log LDA score was settled on 2.0. (**D**) α-diversity measured by the Shannon and Simpson index. (**E**) Principal coordinates analysis (PCoA) plot representing β-diversity based on the Bray-Curtis similarity index at the operational taxonomic unit (OTU) level. The percentage of the total variance explained is indicated in parentheses on each axis. * *p* < 0.05 vs. iDILI-nonAC by a non-parametric Kruskal–Wallis test followed by the Mann–Whitney U-Test. iDILI-AC, idiosyncratic drug-induced liver injury caused by the amoxicillin–clavulanate group (n = 6); iDILI-nonAC, idiosyncratic drug-induced liver injury caused by compounds other than the amoxicillin–clavulanate group (n = 18).

In relation to gut microbiota composition, a specific profile related to iDILI-AC was identified, and significant differences at the phylum, family and genus levels were found. Herein, at the phylum level, the iDILI-AC group showed a higher relative abundance of Bacteroidota and a lower relative abundance of Bacillota and Actinomycetota compared to iDILI-nonAC (Figure 6A).

Moreover, an increase in the relative abundance of the *Catenibacterium* genus and a decrease in the Barnesiellaceae family and *Barnesiella*, *Lachnospira* and *Oscillibacter* genera were observed in iDILI-AC patients (Figure 6B). The LDA score showed a positive association between the *Catenibacterium* genus and iDILI-AC (Figure 6C). The Shannon and Simpson index of α-diversity showed a slight reduction related to AC, although no statistical significance was found (Figure 6D). Additionally, regarding α-diversity performed by a PCoA based on the Bray–Curtis index at the OTU level, the iDILI-nonAC group showed higher dispersion of bacterial communities, whereas the iDILI-AC bacterial communities tended to group along PC2 (PERMANOVA *p* = 0.7381) (Figure 6E). 

**Figure 7:**
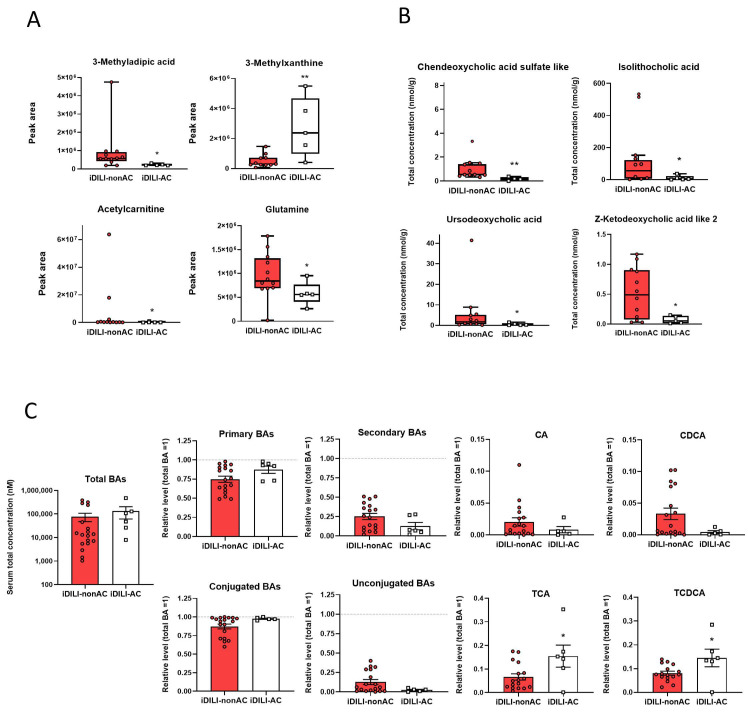
Differences in patients with idiosyncratic drug-induced liver injury caused by amoxicillin–clavulanate in fecal metabolomic profile and fecal bile acid quantification compared with iDILI-nonAC. (**A**) Boxplots representing non-targeted metabolites expressed as the peak area of detection. (**B**) Boxplots representing the concentration of fecal bile acids. (**C**) Total concentration and relative levels of bile acids in serum. The dashed line represents total bile acid levels relativised to one. * *p* < 0.05; ** *p* < 0.01 vs. iDILI-nonAC by a non-parametric Kruskal–Wallis test followed by the Mann–Whitney U-Test Wallis test. Serum bile acids were analyzed by a one-way ANOVA test and data are presented as mean ± SEM. BAs, bile acids; CA, cholic acid; CDCA, chenodeoxycholic acid; iDILI-AC, idiosyncratic drug-induced liver injury caused by the amoxicillin–clavulanate group (Figure 6A,B: n = 5; Figure 6C: n = 6); iDILI-nonAC, idiosyncratic drug-induced liver injury caused by compounds other than the amoxicillin–clavulanate group (Figure 6A,B: n = 12; Figure 6C: n = 17); TCA, taurocholic acid; TCDCA, taurochenodeoxycholic acid.

The metabolomic analysis also showed a particular profile associated with iDILI-AC, as a higher detection of 3-methylxanthyne and a lower detection of acetylcarnitine, glutamine and 3-methylapidic acid were observed in iDILI-AC compared to iDILI-nonAC (Figure 7A).

Additionally, the quantification of BAs in serum and feces also showed a specific pattern related to iDILI-AC. Thus, a reduction in the fecal concentration of chenodeoxycholic acid sulfate like, isolithocholic acid, ursodeoxycholic acid and Z-ketodeoxycholic acid like 2 was observed in iDILI-AC (Figure 7B). Moreover, although serum total BAs levels were similar between iDILI-AC and iDILI-nonAC, the relative levels of primary BAs were slightly higher, while relative levels of secondary BAs were lower in the iDILI-AC group compared to iDILI-nonAC. Furthermore, almost all BAs in the iDILI-AC group were conjugated, whereas relative levels of unconjugated species were close to zero. Consequently, some BA species, such as primary BAs taurocholic acid (TCA) and taurochenodeoxycholic acid (TCDCA), were significantly higher in the iDILI-AC than in the iDILI-nonAC group (Figure 7C).

## 3. Discussion

To the best of our knowledge, this is the first study that unravels the specific compositional and functional gut microbial profile in iDILI and iDILI-AC patients. However, there are some limitations that should be considered. First, as a consequence of iDILI having a low worldwide incidence, the number of samples in some groups is scarce. Second, due to the low prevalence of the disease, we included a wide age range of patients. Third, fecal material was not available to perform targeted and untargeted metabolomic analysis in all the patients. We were not able to collect more fecal material due to the importance of the time frame for sample recruitment during iDILI onset. Fourth, the influence that antibiotics themselves have on the composition of the gut microbiota, regardless of the iDILI condition, was not considered in this study. Lastly, the link between the gut microbiome and iDILI and, particularly, iDILI-AC development could not be explained due to the lack of a mechanistic relationship. 

Multiple factors are involved in iDILI onset and progression, with amoxicillin–clavulanate being one of the main causative drugs. Alterations in gut microbiota composition may play a key role in the development of the disease due to its close connection to liver and metabolic dysfunction as well as to BA homeostasis. For these reasons, deepening the understanding of the link between gut microbiota and iDILI could enable the identification of novel susceptibility (idiosyncratic) factors and provide useful insights into the prevention and treatment of this disease. In this innovative study, we unraveled the existence of particular gut microbiota profiles associated with iDILI and iDILI-AC. In addition, specific alterations associated with each group in the fecal metabolome and the BA profile in serum and feces was detected. Correlation analyses performed showed that some of these changes are likely linked to modifications in gut microbiota composition, reinforcing the hypothesis that gut microbiota could have a relevant role in the onset and progression of iDILI. 

Gut microbiota composition analysis confirmed the presence of a specific gut microbial profile associated with the iDILI. In fact, a Venn diagram at the OTU level determined that 40% of OTUs detected in the iDILI group were not shared with the C and H groups. This microbial signature was characterized by an increase in the relative abundance of the *Sutterellaceae* family and the *Alloprevotella* and *Anaerotruncus* genera, together with a decrease in *Eubacterium* and *Parasutterella*. High levels of *Alloprevotella* have been observed in patients with bile reflux, a process that is directly linked with intestinal inflammation [21]. Related to that, an increase in the main pro-inflammatory markers such as TNF-α and IL-6, together with an increase in the relative abundance of *Alloprevotella,* were detected in a mice model of ulcerative colitis [22], suggesting altogether the possible role of this genus in inflammatory status. Moreover, the *Eubacterium* genus correlated positively with the homeostasis of gut microbiota composition in older persons, but negatively with inflammatory markers [23]. *Anaerotruncus*, which was augmented in the iDILI group, increased in a murine model of non-alcoholic fatty liver disease (NAFLD)-hepatocellular carcinoma [24]. Altogether, these results could point to the existence of a specific gut microbiota profile in iDILI, mainly related to an increased inflammatory status. Furthermore, a common microbial pattern was observed among the iDILI and H groups, probably linked to hepatic damage. Herein, a reduction in the relative abundance of the Barnesiellaceae and Clostridiaceae families and the *Barnesiella*, *Clostridia UCG-014* and *Monoglobus* genera were identified in both the iDILI and H groups. *Barnesiella* was positively correlated with intestinal immunomodulatory capacity [25], while *Clostridia UCG-014* was positively associated with the presence of intestinal tight junction proteins in patients with breast cancer-related fatigue [26]. Moreover, *Monoglobus* was reported to correlate with an enhancement of systemic inflammation in humans [27]. Based on these results, a reduction in these genera could denote a higher predisposition of the iDILI and H groups to a disruption of gut homeostasis that could favor inflammation and gut permeability and worsen liver damage. 

To identify the functional profile related to the iDILI microbial signature, a fecal metabolomic analysis was performed, observing a significant reduction in several fecal metabolites in iDILI patients compared to both the H and C groups. Herein, dodecanedioic acid decreased in our iDILI group. It has been described to have anti-inflammatory and hepatoprotective effects in a murine animal model of liver inflammation [28]. Moreover, N-acetylglutamic acid, a fecal metabolite that was also reduced in our iDILI patients, activates the carbamoyl phosphate synthetase 1 in urea metabolism to obtain carbamoyl phosphate. This enzyme, although it is frequently found in bile, has been identified in systemic circulation after hepatic failure, providing an anti-inflammatory effect against acetaminophen-induced acute liver injury [29]. Additionally, pyridoxamine metabolite, reduced in the iDILI group, was administered to a rat model of hepatic fibrosis, leading to higher levels of glutathione and an improvement in the oxidative status [30]. Even though no direct associations of these metabolites to iDILI have been previously described, its depletion could lead to an increased inflammatory and oxidative stress status that worsens this condition and favors iDILI onset and progression.

Due to the important link between BA metabolism and DILI development, serum and fecal analyses of BAs were performed. In our study, iDILI patients showed a significantly higher concentration of serum total BAs, a well-established marker of liver injury for both hepatocellular and hepatobiliary damage [19]. Additionally, both the H and iDILI groups presented lower levels of secondary BAs-derived metabolites in feces. Related to these results, secondary BAs have a crucial role in health status, such as preventing *Clostridium difficile* overgrowth and hepatocellular carcinoma and enhancing immunity as well as metabolic responses [31]. In this sense, it has been observed that secondary BAs were significantly reduced in patients with cirrhosis [32] and ulcerative colitis [33]. Thus, this reduction in serum and fecal concentration of secondary BAs could be related to a reduction in the capacity to deconjugate and metabolize these compounds by the specific gut microbiota observed in iDILI.

With the purpose of identifying possible hallmarks associated with iDILI, a correlation analysis was performed. Herein, the Barnesiellaceae family and the *Barnesiella* genus were negatively correlated with serum-conjugated BAs and positively correlated with fecal secondary BAs and their derived metabolites. Additionally, the *Barnesiella* genus also showed a negative correlation with serum primary BA levels. These results are in accordance with the reported bile salt hydrolase (BSH) activity of the *Barnesiella* genus, an essential enzyme for BAs deconjugation [34] and consequent secondary BAs formation. Moreover, the *Barnesiella* and *Clostridia UCG014* genera positively correlated with dodecanedioic acid, N-acetylglutamic acid and pyridoxamine fecal metabolites. As previously described, both genera and fecal metabolite abundances were drastically diminished in iDILI patients, suggesting the existence of a particular functional and compositional gut microbiota signature related to iDILI that could be involved in the development of the disease. 

Amoxicillin–clavulanate administration is the main cause of iDILI in Europe and the United States. Therefore, in this study, we also wanted to unravel specific iDILI-AC features to identify possible disease hallmarks. Herein, the particular gut microbiota profile identified in iDILI-AC patients compared to the iDILI-nonAC group was characterized by an increase in the relative abundance of the *Catenibacterium* genus and a reduction in the Barnesiellaceae family and the *Barnesiella* and *Lachnospira* genera. The correlation analysis showed a positive association between iDILI-AC and *Catenibacterium*, a genus that has also been related to hepatic encephalopathy [35] and to high animal fat diet consumption in humans [36]. Additionally, Barnesiellaceae and *Barnesiella*, whose abundance was also diminished in the iDILI-nonAC group compared to healthy subjects, showed a stronger reduction in iDILI-AC patients. Furthermore, the *Lachnospira* genus, reduced in iDILI-AC compared to iDILI-nonAC, has been positively correlated with serum antioxidant activities and negatively correlated with inflammatory cytokines in mice [37]. 

Regarding the iDILI-AC fecal metabolome profile, the concentration of 3-methylxanthyne increased, whereas glutamine decreased compared to the iDILI-nonAC group. Higher concentrations of 3-methylxanthyne are able to inhibit human cytochrome P450 enzyme 1A2 activity, one of the most important hepatic enzymes involved in drug metabolism [38]. In fact, the expression of this enzyme could be modified by AC in humans [39], suggesting a possible link between AC and the increased concentration of this metabolite. Moreover, glutamine, diminished in iDILI-AC, is a potential antioxidant and is directly involved in glutathione production [40,41], as well as in the maintenance of gut barrier integrity [42]. According to that, an in vitro study carried out by Petrov et al. (2021) in human hepatocytes described that AC administration and, especially, clavulanate administration produced a depletion of glutathione and downregulation of nuclear factor erythroid 2–related factor 2, a major inductor of antioxidant genes [43]. Therefore, glutamine depletion could be related to the deterioration of the oxidative status in iDILI-AC and, subsequently, a key mechanism related to the onset and progression of the disease.

Finally, although serum BA profiles of the iDILI-nonAC and iDILI-AC groups showed the same trend, primary conjugated BAs TCA and TCDA were found to increase in the serum of iDILI-AC patients, in line with their reduced Barnesiellaceae and *Barnesiella* BSH deconjugating activity. In consonance, fecal secondary BAs (chenodeoxycholic acid sulfate like, isolithocholic acid, ursodeoxycholic acid (UDCA) and Z-ketodeoxycholic acid like 2, which are produced after primary BA deconjugation, showed a significant reduction in iDILI-AC patients. Among them, UDCA is a secondary BA that has been used to ameliorate cholestatic liver injury in iDILI and iDILI-AC patients [44,45,46].

The most frequent liver injury pattern in iDILI-AC patients was the cholestatic type. Cholestasis is associated with impaired bile flow and increased BA levels, which could reach detrimental concentrations of hepatocytes. Our results suggest that some patients on AC may have reduced levels of Barnesiellaceae and *Barnesiella* (among others) and, consequently, have decreased BA deconjugation and reduced secondary BA synthesis and fecal excretion. All these lead to higher circulating BA levels, a condition that may make these patients more susceptible to cholestatic liver damage by AC.

As the main conclusions of our study, our results showed the existence of a distinct gut microbial profile, together with a specific fecal metabolome and a certain BA pattern in iDILI and, particularly, in iDILI-AC patients that could indicate a relationship between the onset and progression of the disease and gut microbiota functionally. However, more studies that enhance the mechanistic explanation of the role of the gut microbiota in iDILI and iDILI-AC are needed to elucidate the existence of possible disease hallmarks and therapeutic targets.

The findings observed in this novel and descriptive study contribute to the discovery of certain microbial and metabolomic biomarkers that could become feasible early predictors of the disease. The detection of valid biomarkers would help to manage the early diagnosis of iDILI, a crucial step to prevent a worsening of the disease state. As a consequence, it could directly influence the improvement not only of the progression of this disease but also of the health economics. Moreover, these results could support future studies focused on unraveling the role of gut microbiota in the onset and progression of iDILI and iDILI-AC, an as yet unknown field of study. 

## 4. Materials and Methods

### 4.1. Participants and Ethical Approval

A total of 46 patients, aged between 28 and 89 years old, all Caucasians, were included in the study and were divided into three experimental groups: 10 healthy control patients (C group), 12 patients with non-iDILI acute hepatitis as a hepatic damage control group (H group); and 24 iDILI patients caused by different compounds (iDILI group). In order to study the effect of AC, the iDILI group was divided into two subgroups: 18 patients with iDILI caused by compounds other than AC (iDILI-nonAC group) and 6 patients with iDILI caused by amoxicillin–clavulanate (iDILI-AC group) (Figure 1). All the subjects were recruited by the Digestive Service of Complejo Asistencial Universitario de León (CAULE) and Hospital Universitario Virgen de la Victoria in Malaga. All patients were biochemically and demographically characterized (Table 2).

iDILI classification was based on the causative drug and the type of liver damage using the ratio (R) between alanine aminotransferase/alkaline phosphatase activities after being divided by the upper limit normal (ULN) (ALT÷ULN/ALP÷ULN) determined in the first biochemical analysis of each subject. According to this ratio, tissue liver injury was classified into hepatocellular (R ≥ 5), cholestatic (R ≤ 2), or mixed (2 < R < 5), as described elsewhere [47]. iDILI diagnosis criteria were based on Clinical Practice Guidelines described by the European Association for the Study of the Liver (EASL), described elsewhere [48]. Causality assessment between the hepatic damage and the suspected causative agent and iDILI classification was performed by using the Roussel Uclaf Causality Assessment Method (RUCAM) developed by the Council for International Organizations of Medical Science (CIOMS) [49].

Inclusion criteria for iDILI were (1) having been exposed to a drug, herbal product or dietary supplement within 6 months prior to clinical examination; (2) iDILI biochemical criteria were [ALT] ≥5 X ULN, [ALP] ≥2 X ULN, or ALT ≥3 X ULN together with total bilirubin levels [TBL] >2 X ULN [47]; (3) having a previous biochemical analysis of liver function within normal or almost normal values. All iDILI cases were at least *possible* by applying RUCAM. Inclusion criteria for non-iDILI acute hepatitis were presenting abnormal values of biochemical parameters associated with acute hepatitis without a related hepatotoxic agent, presenting transaminases 5 times higher than ULN. Patients presenting DILI not caused by a standard therapeutic dose were excluded from the study. Healthy subjects had a similar age range to the other groups, were not on any antibiotic treatment for at least one month prior to sample collection, and presented physiological levels of liver injury-related biochemical parameters. 

The study was conducted according to the guidelines outlined in the Declaration of Helsinki, and all procedures involving human subjects were approved by the local Ethics Committees. All patients gave informed written consent before participating in the study.

### 4.2. Sample Collection

Blood and serum samples were collected to determine clinical hematological and biochemical parameters (Figure 1) during the first 24 h after liver injury detection and the withdrawal of the causative drug. Glucose, urea, creatinine, aspartate aminotransferase (AST), alanine aminotransferase (ALT), alkaline phosphatase (ALP), gamma-glutamyl transferase (GGT), total bilirubin (TBL), albumin, platelets, leukocytes, neutrophils, the International Normalized Ratio (INR) and the serum BA profile were analyzed.

Moreover, fresh stool samples were collected from each patient during the first week after liver injury detection. All the fecal samples were homogenized and aliquoted as follows: 1–2 g of fresh stool was preserved at room temperature in Stool Nucleic Acid Collection and Preservation Tubes (Norgen Biotek, Thorold, ON, Canada) for metagenomic analysis, and 2 g was frozen in less than 1 h at −80 °C until its further analysis (Figure 1). Variations in the number of patients included in each analysis were caused by the impossibility of sample recruitment or a limited amount. 

### 4.3. Fecal Metagenomic Analysis

Total bacterial genomic DNA was isolated from fecal samples using the QIAamp PowerFecal Pro DNA Kit (Qiagen, Hilden, Germany), according to the manufacturer’s instructions. The lysis step was performed using PowerBead Pro Tubes (Qiagen, Hilden, Germany) and a bead mill homogenizer (BeadMill 24, Fisherbrand^TM^, Fisher Scientific, Hampton, NH, USA). Eluted DNA concentration was determined using a NanoDrop-1000 spectrophotometer (NanoDrop Technologies, Wilmington, NC, USA). DNA samples were stored at −20 °C until the analysis. 

Amplification of the 16S rRNA V3-V4 hypervariable region was performed by PCR, as previously described [50]. The PCR assay was carried out in triplicate for each sample and purified with the Wizard^®^ Genomic DNA Purification kit (Promega, Madison, WI, USA), according to the manufacturer’s instructions. 

Gut microbiota composition analysis was performed by the company STAB VIDA (Caparica, Portugal). The resulting amplicons were cleaned, quantified and sequenced on an Illumina MiSeq platform using MiSeq Reagent Kit v3 and 300 bp paired-end. The FastQC software (release 0.12.0) was used to check quality control post-sequencing [51]. The analysis of the generated raw sequence data was carried out using Quantitative Insights into Microbial Ecology software (QIIME2 version 2022.2 [52], and reads were denoised using the DADA2 plugin [53], including trimming and truncating low-quality regions, dereplicating the reads and filtering chimeras. Reads were organized in operational taxonomic units (OTUs) and classified by taxon using a fitted classifier, trained by the SILVA (release 138 QIIME) database, with a clustering threshold of 99% similarity. For classification purposes, only OTUs containing at least 10 sequence reads were considered as significant.

### 4.4. Fecal Metabolomic Analysis

Non-targeted metabolomic analysis of semi-polar compounds was carried out by MS-Omics (Caparica, Portugal) using a Thermo Scientific Vanquish liquid chromatography (LC) coupled to an Orbitrap Exploris 240 mass-spectrometry (MS) (Thermo Fisher Scientific Waltham, MA, USA). The ionization source was an electrospray ionization interface. Analysis was carried out in positive and negative ionization modes under polarity switching. The ultra-performance liquid chromatography (UPLC) procedure was performed based on a slight modification of the protocol described by Doneanu et al. [54]. Compound Discoverer 3.3 from Thermo Scientific was used to extract peak areas. The identification of the different compounds was performed at four different levels based on the retention times, accurate mass, and tandem mass spectrometry (MS/MS) spectra.

### 4.5. Fecal Short-Chain Fatty Acids (SCFAs) Quantification

SCFAs analysis was carried out by MS-Omics (Vedbaek, Denmark). First, deuterium-labeled internal standards were added to the samples, which were previously acidified using hydrochloride acid. The analysis was performed using a high polarity column (7890B, Agilent, Santa Clara, CA, USA) coupled with a quadrupole detector (7890B, Agilent, Santa Clara, CA, USA). The system was controlled by ChemStation (Agilent, Santa Clara, CA, USA). Prior to being imported and analyzed in MATLAB R2021b (Mathworks, Inc., Natick, MA, USA), using the PARADISe program described by Johnsen et al. [55], raw data were transformed to netCDF format using Chemstation (Agilent, Santa Clara, CA, USA).

### 4.6. Fecal Bile Acid Quantification

Fecal BA quantification was carried out by MS-Omics (Vedbaek, Denmark). First, an internal standard was added to the samples, which were diluted five times. The analysis was performed using a UPLC system (Vanquish, Thermo Fisher Scientific, Waltham, MA, USA) coupled with a high-resolution quadrupole-orbitrap mass spectrometer (Q Exactive™ HF Hybrid Quadrupole-Orbitrap, Thermo Fisher Scientific, Waltham, MA, USA). Analysis was performed in the negative ionization mode using an electrospray ionization interface. The chromatographic separation of BAs was carried out on a Waters Acquity HSS T3 1.8 μm 2.1 × 150 mm (Waters, Milford, MA, USA). The thermostat in the column was set at 30 °C. For ammonium acetate (10 mmol/L) and methanol, acetonitrile (1:1) constituted the mobile phases. The elution of BAs was performed by increasing the methanol: acetonitrile in ammonium acetate from 45% to 100% for 16 min. The flow rate was 0.3 mL/min. Peak areas were extracted using Skyline version 22.2 (MacCoss Lab Software, Seattle, WA, USA), and the identification of compounds was based on the accurate mass and retention time of authentic standards.

### 4.7. Serum Bile Acid Quantification

Serum BAs were quantified in the Analytical Unit, Core Facility, IIS Hospital La Fe in Valencia (Spain). A UPLC-multivariate reaction monitoring-mass spectrometry (UPLC-MRM-MS) method was used as described previously [56]. Internal standards were added to 50 µL of serum. Proteins were then precipitated, followed by the drying and reconstitution of supernatants in 50 µL of methanol:water (50:50, *v*/*v*). An Acquity UPLC system (Waters, Wilmslow, UK) outfitted with an Acquity UPLC BEH C18 column (1.7 m, 2.1 × 100 mm; Waters, Milford, MA, USA) was used to analyze the samples. A Waters Xevo TQ-S mass spectrometer with an ESI source operating in the negative-ion mode was used to conduct the MS study. Using five additional deuterated BAs as internal standards, this technique enabled the measurement of 12 unconjugated, 8 glycine-conjugated, and 11 taurine-conjugated BAs in a single analytical run. 

### 4.8. Statistical Analysis

A normality test was performed for all samples. For biochemical analysis, a one-way ANOVA test for multiple pairwise comparisons was performed considering a *p* < 0.05 for significant differences. Results were expressed as the mean ± standard error of the mean (SEM). For microbial and metabolomic data, statistical significance was determined by a non-parametrical Kruskal–Wallis test followed by the Mann–Whitney U-Test with *p* < 0.05. The significance of the different factor’s effect on the bacterial community distribution was obtained using permutational multivariate analysis of variance (PERMANOVA). Spearman’s correlation coefficient was performed to evaluate correlations between biochemical, microbial and metabolomic data. Statistical analyses were performed using SPSS 26.0 software for Windows (Chicago, IL, USA), R software version 4.2.3 (R-project, Vienna, Austria) and GraphPad Prism software version 8.0.2.

## Figures and Tables

**Table 1:** Hematological and biochemical parameters from patients.

	C	iDILI	H	*p* Value
Glucose (mg/dL)	88 ± 4	109 ± 6	111 ± 9	0.184
Urea (mg/dL)	33 ± 3	45 ± 7	36 ± 8	0.275
Creatinine (mg/dL)	0.80 ± 0.03	0.89 ± 0.08	0.98 ± 0.15	0.930
ALT (U/L)	22 ± 3	741 ± 200 ***^#^	1349 ± 282 ***	<0.001
AST (U/L)	22 ± 2	487 ± 142 ***^##^	1048 ± 236 ***	<0.001
ALP (U/L)	70 ± 6	246 ± 33 **	192 ± 24 ***	0.004
GGT (U/L)	22 ± 4	283 ± 48 ***	473 ± 70 ***	<0.001
TBL (mg/dL)	0.50 ± 0.05	5.59 ± 1.22 **	8.90 ± 2.27 ***	0.002
Albumin (g/dL)	4.71 ± 0.04	3.58 ± 0.13 ***	3.66 ± 0.23 *	0.006
Platelets (10^3^/µL)	230 ± 17	242 ± 16	220 ± 27	0.631
Leukocytes (10^3^/µL)	5.5 ± 0.3	6.9 ± 0.6	8.2 ± 1.1	0.160
Neutrophils (10^3^/µL)	2.9 ± 0.2	3.6 ± 0.3 ^#^	5.9 ± 0.9 *	0.027
INR	0.98 ± 0.03	1.30 ± 0.17	1.38 ± 0.17 **	0.024

Data are presented as mean ± SEM. C, healthy control group (n = 10); iDILI, idiosyncratic drug-induced liver injury group (n = 24); H, non-iDILI acute hepatitis group (n = 12); AST, aspartate aminotransferase; ALP, alkaline phosphatase (Upper normal limit = 130 U/L); ALT, aspartate aminotransferase; GGT, gamma-glutamyl transferase (Upper normal limit = 61 U/L); INR, International Normalized Ratio; TBL, total bilirubin levels. * *p* < 0.05; ** *p* < 0.01; *** *p* < 0.001 vs. C; ^#^ *p* < 0.05; ^##^ *p* < 0.01 vs. H by one-way ANOVA test. Outliers were identified and removed before statistical analysis.

**Table 2:** Demographical and clinical characterization of patients.

Demographic Parameters	Data
Age, mean ± SEM	58 ± 2
Sex, n (%)	
Men	19 (43)
Women	27 (57)
**Samples recruitment, n/total (%)**	**Data**
Healthy controls	10/46 (21.7)
non-iDILI acute hepatitis	12/46 (26.1)
iDILI	24/46 (52.2)
iDILI-nonAC	18/24 (75)
iDILI-AC	6/24 (25)
**iDILI liver damage, n/total (%)**	**Data**
Hepatocellular	9/24 (37.5)
Cholestatic	11/24 (45.8)
Mixed	4/24 (16.7)
**iDILI causal agent, n/total (%)**	**Data**
Antibiotics	14/24 (58.3)
Amoxicillin–clavulanate	6/14 (43)
NSAIDs	5/24 (20.8)
Statins	3/24 (12.5)
Antifungals	1/24 (4.2)
Chemotherapeutics	1/24 (4.2)
**Non-iDILI acute hepatitis causal agent, n/total (%)**	**Data**
Autoimmune hepatitis	9/12 (75)
Hepatitis C	1/12 (8.3)
Hepatitis B	2/12 (16.7)

DILI, drug-induced liver injury; iDILI, idiosyncratic drug-induced liver injury; iDILI-AC, idiosyncratic drug-induced liver injury caused by amoxicillin–clavulanate; iDILI-nonAC, idiosyncratic drug-induced liver injury caused by compounds other than amoxicillin–clavulanate; n, number of samples; NSAIDs, non-steroidal anti-inflammatory medications; SEM, standard error of the mean.

## Data Availability

Data are contained within the article.

## Supplementary Materials

The supporting information can be downloaded at: https://www.mdpi.com/article/10.3390/ijms25136863/s1.
