# Supplementary material for: Idiosyncratic Drug-Induced Liver Injury and Amoxicillin–Clavulanate: Spotlight on Gut Microbiota, Fecal Metabolome and Bile Acid Profile in Patients
Source: Int J Mol Sci. 2024 Jun 22;25(13):6863. doi: 10.3390/ijms25136863 (PMC11241776; doi:10.3390/ijms25136863)
Supplement: Supplementary file 1 [file ijms-25-06863-s001.zip › ijms-3043067-supplementary.pdf]

SUPPLEMENTARY INFORMATION

Idiosyncratic Drug-Induced Liver Injury and Amoxicillin–Clavulanate: Spotlight on Gut Microbiota, Fecal Metabolome and Bile Acid Profile in Patients

Sara Román-Sagüillo [1,†], Raisa Quiñones Castro [2,†], María Juárez-Fernández [1,3], Polina Soluyanova [4,5], Camilla Stephens [3,6], Mercedes Robles-Díaz [3,6], Francisco Jorquera Plaza [1,2,3], Javier González-Gallego [1,3], Susana Martínez-Flórez [1], María Victoria García-Mediavilla [1,3], Esther Nistal [1,3], Ramiro Jover [3,4,5] and Sonia Sánchez-Campos [1,3,*]

[1] Instituto Universitario de Biomedicina (IBIOMED), Universidad de León, 24071 León, Spain; sroms@unileon.es (S.R.-S.); mjuarf@unileon.es (M.J.-F.); fjorqueraplaza@gmail.com (F.J.P.); jgonga@unileon.es (J.G.-G.); smarf@unileon.es (S.M.-F.); mvgarm@unileon.es (M.V.G.-M.); menisg@unileon.es (E.N.)
[2] Servicio de Aparato Digestivo, Complejo Asistencial Universitario de León, 24008 León, Spain; rquinones@saludcastillayleon.es
[3] Centro de Investigación Biomédica en Red de Enfermedades Hepáticas y Digestivas (CIBERehd), Instituto de Salud Carlos III, 28029 *Madrid*, Spain; cstephens@uma.es (C.S.); mercedesroblesdiaz@hotmail.com (M.R.-D.); ramiro.jover@uv.es (R.J.)
[4] Unidad Mixta de Investigación en Hepatología Experimental, IIS Hospital La Fe, 46026 Valencia, Spain; polina.soluyanova@uv.es
[5] Departamento de Bioquímica y Biología Molecular, Universidad de Valencia, 46010 Valencia, Spain
[6] Unidad de Gestión Clínica de Aparato Digestivo y Servicio de Farmacología Clínica, Instituto de Investigación Biomédica de Málaga-IBIMA plataforma BIONAND, Hospital Universitario Virgen de la Victoria, Facultad de Medicina, Universidad de Málaga, 29010 Málaga, Spain
* Correspondence: ssanc@unileon.es; Tel.: +34-987291266
† These authors contributed equally to this work.

SUPPLEMENTARY INFORMATION

Supplementary Table S1 2

Supplementary Figure S1..3

Supplementary Table S1. Hematological and biochemical parameters from iDILI-nonAC and iDILI-AC patients.

	**iDILI-nonAC**	**iDILI-AC**	***p* value**
Glucose (mg/dL)	104 ± 7	126 ± 14	0.104
Urea (mg/dL)	41 ± 8	54 ± 11	0.052
Creatinine (mg/dL)	0.89 ± 0.10	0.91 ± 0.1	0.31
ALT (U/L)	696 ± 154	878 ± 699	0.31
AST (U/L)	546 ± 181	310 ± 174	0.871
ALP (U/L)	236 ± 42	273 ± 52	0.494
GGT (U/L)	260 ± 49	340 ± 123	1
TBL (mg/dL)	4.6 ± 1.5	8.37 ± 1.92	0.137
Albumin (g/dL)	3.57 ± 0.17	3.58 ± 0.22	1
Platelets (10^3/μL)	255 ± 16	204 ± 36	0.177
Leukocytes (10^3/μL)	6.9 ± 0.7	6.8 ± 0.9	0.919
Neutrophils (10^3/μL)	3.5 ± 0.3	3.8 ± 0.6	1
INR	1.36 ± 0.23	1.16 ± 0.1	0.747

Data are presented as mean ± SEM. C; iDILI, idiosyncratic drug-induced liver injury group; iDILI-AC, idiosyncratic drug-induced liver injury caused by amoxicillin-clavulanate group (n = 6); iDILI-nonAC, idiosyncratic drug-induced liver injury caused by compounds other than amoxicillin-clavulanate group (n = 18); AST, aspartate aminotransferase; ALP, alkaline phosphatase; ALT, aspartate aminotransferase; GGT, gamma-glutamyl transferase; INR, International Normalized Ratio; TBL, total bilirubin levels. One-way ANOVA test was performed. Outliers were identified and removed before statistical analysis.

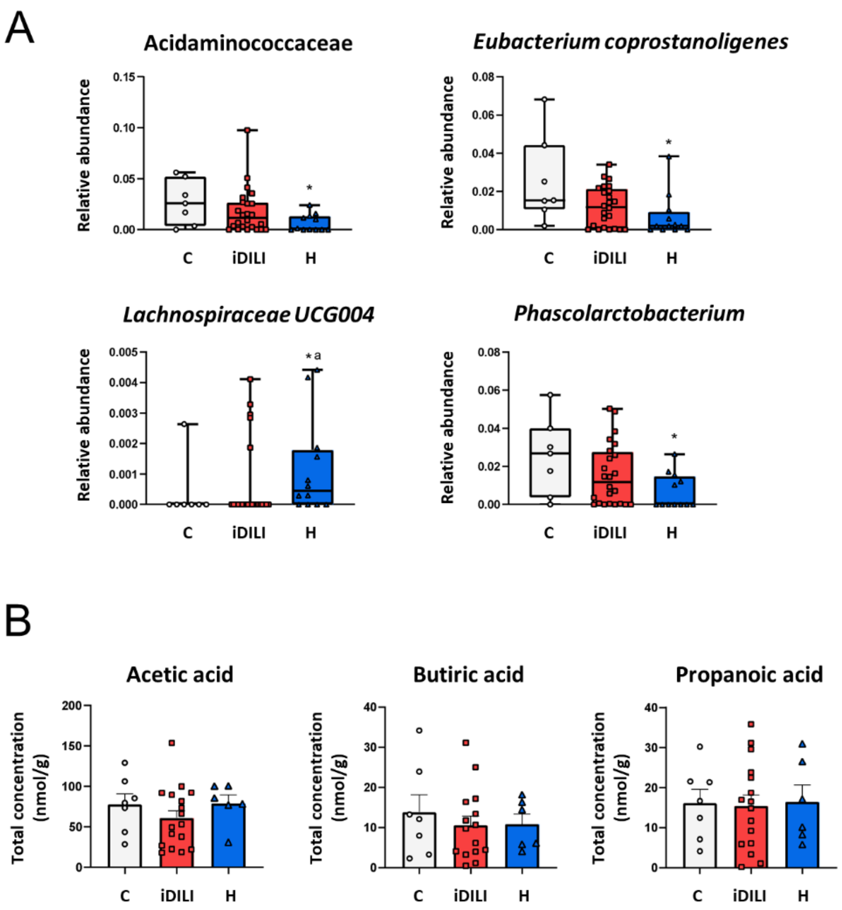

Supplementary Figure S1. (A) Differences in gut microbiota composition associated with non-iDILI acute hepatitis (B) Principal fecal SCFAs. C, healthy control group (n = 7); iDILI, idiosyncratic drug-induced liver disease group (S1A: n = 24; S1B: n = 17); H, non-iDILI acute hepatitis group (S1A: n = 12; S1B: n = 6). *$p < 0.05$ vs. C; [a]$p < 0.05$ vs. iDILI by non-parametric Kruskal-Wallis test followed by Mann-Whitney U-Test.
